# Motor Sequence Learning and Consolidation in Unilateral *De Novo* Patients with Parkinson’s Disease

**DOI:** 10.1371/journal.pone.0134291

**Published:** 2015-07-29

**Authors:** Xiaojuan Dan, Bradley R. King, Julien Doyon, Piu Chan

**Affiliations:** 1 Department of Neurobiology and Neurology, Key Laboratory of Ministry of Education on Neurodegenerative Disorders, Beijing Key Laboratory on Parkinson’s Disease, Xuanwu Hospital of Capital Medical University, Beijing, China; 2 Functional Neuroimaging Unit, Department of Psychology, Université de Montréal, Montréal, Quebec, Canada; 3 Parkinson’s Disease Center of Beijing Institute for Brain Disorders, Beijing, China; University of Chicago, UNITED STATES

## Abstract

Previous research investigating motor sequence learning (MSL) and consolidation in patients with Parkinson’s disease (PD) has predominantly included heterogeneous participant samples with early and advanced disease stages; thus, little is known about the onset of potential behavioral impairments. We employed a multisession MSL paradigm to investigate whether behavioral deficits in learning and consolidation appear immediately after or prior to the detection of clinical symptoms in the tested (left) hand. Specifically, our patient sample was limited to recently diagnosed patients with pure unilateral PD. The left hand symptomatic (LH-S) patients provided an assessment of performance following the onset of clinical symptoms in the tested hand. Conversely, right hand affected (left hand asymptomatic, LH-A) patients served to investigate whether MSL impairments appear before symptoms in the tested hand. LH-S patients demonstrated impaired learning during the initial training session and both LH-S and LH-A patients demonstrated decreased performance compared to controls during the next-day retest. Critically, the impairments in later learning stages in the LH-A patients were evident even before the appearance of traditional clinical symptoms in the tested hand. Results may be explained by the progression of disease-related alterations in relevant corticostriatal networks.

## Introduction

Motor sequence learning (MSL) involves integrating the temporal structuring of a series of actions into a coherent unit and is thought to follow several phases [1–4]. A rapid learning phase is characterized by substantial behavioral improvements during the initial training session. Smaller improvements subsequently emerge over extended practice and an intermediate consolidation phase occurs between practice sessions, during which the motor memory is processed offline without any further training. Whereas the initial learning stage is dependent on widespread cortical and subcortical structures, both the late learning and consolidation phases are predominantly a function of cortico-striatal and cortico-hippocampal networks [4–9]. Given the substantial striatal degeneration evident in Parkinson’s disease (PD) and the critical role of corticostriatal circuitry in MSL and consolidation, it is likely that PD adversely affects these learning and memory processes.

Previous research investigating MSL in PD has predominantly focused on the initial learning phase [10–22]. The results have been largely inconsistent with respect to whether patients with PD demonstrate deficits (see [22] for review). The inconsistent findings are likely the result of several factors, including methodological variations in the task employed (e.g., implicit vs. explicit, bimanual vs. unimanual, etc.), the influence of dopaminergic medication [16,19,22–24] as well as the stage of the disease in the sample of participants with PD [16,20,25]. Relative to initial learning, however, less is known about the consolidation and extended practice phases. Patients with PD appear to show similar overnight memory consolidation as healthy controls [21] but exhibit impairments during the extended practice or automatization phases [21,25–28]. Such a deficit is supported by research indicating that the contribution of the corticostriatal system increases as a function of learning [6] and that with continued practice, there is a shift in activation from the associative to the sensorimotor regions of the striatum that are more impacted in PD, particularly during early disease stages [29–31].

As previous research has predominantly included participant samples with early and advanced stages of PD, little is known about the onset of potential behavioral impairments in the learning and consolidation processes. Accordingly, the primary objective of the current study was to investigate whether motor learning and consolidation deficits in patients with PD appear immediately after or prior to the detection of clinical symptoms in the tested hand. To achieve this aim, our patient sample was limited to a unique cohort of recently diagnosed patients with pure unilateral PD. The motor deficits were thus limited to the ipsilateral or contralateral hand used to perform our motor sequence task (left). The left hand affected patients with PD, referred to as left hand symptomatic (LH-S), enabled us to determine if deficits appear immediately following the appearance of the overt clinical symptoms in the tested hand. Conversely, the unilateral, right hand affected patients, referred to as left hand asymptomatic (LH-A), provided a preclinical ‘model’ to investigate whether impairments appear prior to the detection of traditional symptoms in the tested hand. Indeed, the pathologic process of PD—the nigrostriatal dopaminergic deficit—begins well before the appearance of the traditional symptoms used by clinicians for diagnosis [32–36] and previous imaging studies even revealed changes in both the striatum and sensorimotor cortices contralateral to the non-affected hand in patients with PD [37–39].

We asked a cohort of *de novo* patients with pure unilateral PD to complete two sessions (carried out on consecutive days) of a MSL task with the non-dominant left hand. This design allowed us to assess potential deficits across the various learning stages (i.e., initial learning, extended practice and offline consolidation). It was hypothesized that both groups of patients with PD would demonstrate impairments in late learning and consolidation, as these processes are dependent on the corticostriatal circuitry that shows dopaminergic denervation early in the disease process [29–31]. This hypothesized result would suggest that deficits in the extended practice and consolidation phases are evident even before the appearance of traditional clinical symptoms in the tested hand.

## Methods

### 2.1. Subjects

Forty-four *de novo*, drug naive patients with idiopathic, unilateral PD (19 left and 25 right arm affected patients) were recruited for this study. The inclusion of non-medicated patients allowed us to avoid the influence of medication on learning and consolidation in PD [19,23,24]. Patients were recruited from the movement disorder center at Xuanwu Hospital of Capital Medical University in Beijing. Thirty age-matched, healthy controls were recruited from the Beijing Longitudinal Study on Aging community cohort. All subjects were right-handed based on the Edinburgh Handedness Inventory [40]. To control for varying levels of experience on tasks similar to that employed in the current study, participants classified as musicians or professional typists were not permitted to participate. All patients and controls were of Chinese Han ethnicity and reported no known history of drug or alcohol abuse, or any neurologic (other than PD), psychiatric or sleep disorders. Participants who scored <24 on the Mini-Mental State Examination (MMSE) [41] or ≥10 on the Geriatric Depression Scale (GDS; 30 items) [42,43] were excluded. A movement disorder specialist conducted standard clinical evaluations, including the Unified Parkinson’s Disease Rating Scale (UPDRS) and Hoehn and Yahr stage on the patients. Three participants in total (one from each experimental group) were excluded from the analyses due to being outliers on performance on the motor sequence task. Participant characteristics for those included in the analyses are included in Table 1. The Research Ethics Committee of Xuanwu Hospital approved this study and all participants provided written informed consent prior to participation.

**Table 1:** Participant characteristics.

	LH-S	LH-A	Controls
n	18 (8 females)	24 (13 females)	29 (16 females)
Age (yrs)	59.4 ± 7.7	57.7 ± 8.8	61.5 ± 7.4
Education (yrs)	9.5 ± 3.7	10.8 ± 2.9	10.8 ± 2.8
MMSE	27.9 ± 1.9	28.4 ± 1.4	29.1± 1.1
GDS	5.7 ± 2.6	5.8 ± 2.4	3.9 ± 3.1
Disease duration (yrs)	1.3± 1.0	1.6 ± 1.0	N/A
UPDRS I	0.7 ± 1.0	1.2 ± 1.9	N/A
UPDRS II	5.8 ± 2.9	6.4 ± 2.6	N/A
UPDRS III	9.6 ± 3.5	9.4 ± 3.2	N/A
Hoehn and Yahr	1.2 ± 0.2	1.1 ± 0.2	N/A
Tremor	2.7 ± 1.6	2.1 ± 1.4	N/A
Bradykinesia	3.2 ± 2.0	3.7 ± 1.5	N/A
Rigidity	1.9 ± 1.0	2.1 ± 1.5	N/A
Gait / Posture	0.3 ± 0.5	0.3 ± 0.6	N/A
Bulbar abnormalities	1.4 ± 0.9	1.3 ± 0.8	N/A
L-UPDRS III	6.8 ± 2.9	0 ± 0.2	N/A
R-UPDRS III	0 ± 0	6.7 ± 2.0	N/A
PD-dominant	8 tremor; 2 PIGD; 3 intermediate	12 tremor; 2 PIGD; 8 intermediate	N/A

Values are means ± SD. MMSE = Mini Mental State Examination; GDS = Geriatric Depression Scale; UPDRS = Unified Parkinson’s Disease Rating Scale. L-UPDRS III = left side scores of UPDRS part III; R-UPDRS III = right side scores of UPDRS part III. PIGD = postural instability—gait difficulty. PD-dominance was based on method employed in [44].

## 2.2. Experimental procedure

Participation consisted of two experimental sessions separated by approximately 24 hours. The first session included administration of the clinical assessments, the MMSE and GDS as well as the initial training on the motor sequence finger tapping task (see below for task details). The motor sequence task was again administered at the beginning of Session 2. Importantly, to minimize the potential detrimental influence of interference, there were no other experimental procedures administered between the two training sessions of the motor sequence task and participants were instructed to avoid activities involving sequences of finger movements (e.g., typing) throughout participation in the protocol. Furthermore, the two sessions were administered at approximately the same time of day to minimize the influence of circadian effects on task performance.

**Fig 1:**
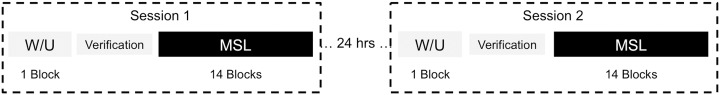
Experimental Design. See text for details of each phase. W/U = warm-up.

The two sessions of finger sequence learning were identical (Fig 1). Participants were seated comfortably in a height-adjustable chair in front of a computer monitor. The left hand of the participants was positioned on a four-button response box located left of the participants’ midlines. In order to afford subjects a brief warm-up period and adaptation to the response box prior to initiating the sequence task, participants simultaneously pressed all four buttons on the response pad with the four fingers of the left hand as fast as possible (Fig 1: Warm-up; W/U). Participants were instructed to start once the fixation cross shown on a computer monitor turned green and to continue until it changed to red. Unbeknownst to the participants, the cross remained green until the participants completed 60 presses (i.e., simultaneous flexion of the four fingers). Next, participants completed an adapted version of the motor sequence finger tapping task [1] employed extensively in previous research [5,9]. Briefly, participants were instructed to use the fingers of their left hand and the corresponding buttons of the response box to perform an explicitly known sequence of finger presses: 4-1-3-2-4, where 4 and 1 correspond to the little and index fingers, respectively. To verify that participants memorized the appropriate sequence of finger movements, they were asked to complete three consecutive sequences slowly and without errors prior to the training session (Fig 1: Verification). Next, participants completed the training portion by performing the sequence as fast and as accurately as possible while the green cross was displayed on the computer monitor (Fig 1: MSL). Participants were instructed to return to the beginning of the sequence if they realized an error was made. The MSL task contained 14 blocks, with each block consisting of 60 key presses (ideally corresponding to 12 correct sequences). In between blocks, a red cross was displayed on the monitor for a duration of 25 seconds during which participants were instructed to rest their hand.

### 2.3. Data analysis

Performance was assessed with measures of movement speed (Block Duration, defined as the time to complete the 60 key presses in each block) and movement accuracy (number of correct sequences in a given block). We also computed an aggregate speed / accuracy measure referred to as Performance Index (PI; Eq 1).

$${\text{PI}}_{\text{x}}{\text{ = exp}}^{\text{-(BLDur/12)}}{\text{*exp}}^{\text{-(Errors/12)}}\text{*}100$$ (1)

where x = blocks of trials

Errors = maximum number of correct sequences (i.e., 12) minus the number of actual correct sequences within each block

Since both speed and accuracy were modulated by practice of the MSL task (see Results below) and in order to increase the interpretability of the data, our conclusions were based predominantly on PI. To control for the small, yet non-significant differences in age among the three groups (see Table 1), all statistical analyses were conducted with age as a covariate. The interactions between the covariate Age and the independent variable Group were checked for each analyses to ensure homogeneity of regression slopes was not violated. Each dependent measure was analyzed with separate 3 (Group) x 14 (Block) mixed model ANCOVAs for each experimental session. Significant Group x Block interactions and Group main effects were followed-up with three different Group (2 levels) x Block (14) ANCOVAs to decompose the effect(s) of interest. The analyses on Sessions 1 and 2 were used to assess the initial learning and extended training phases, respectively. Memory consolidation was assessed by computing offline changes [45,46], defined as the changes from the last 2 blocks of Session 1 to the first 2 blocks of Session 2. Offline changes were analyzed by a one-way (Group) ANCOVA. Significance threshold was set at 0.05 for all contrasts.

## Results

### 3.1. Initial Learning

#### 3.1.1. Movement Speed

**Fig 2:**
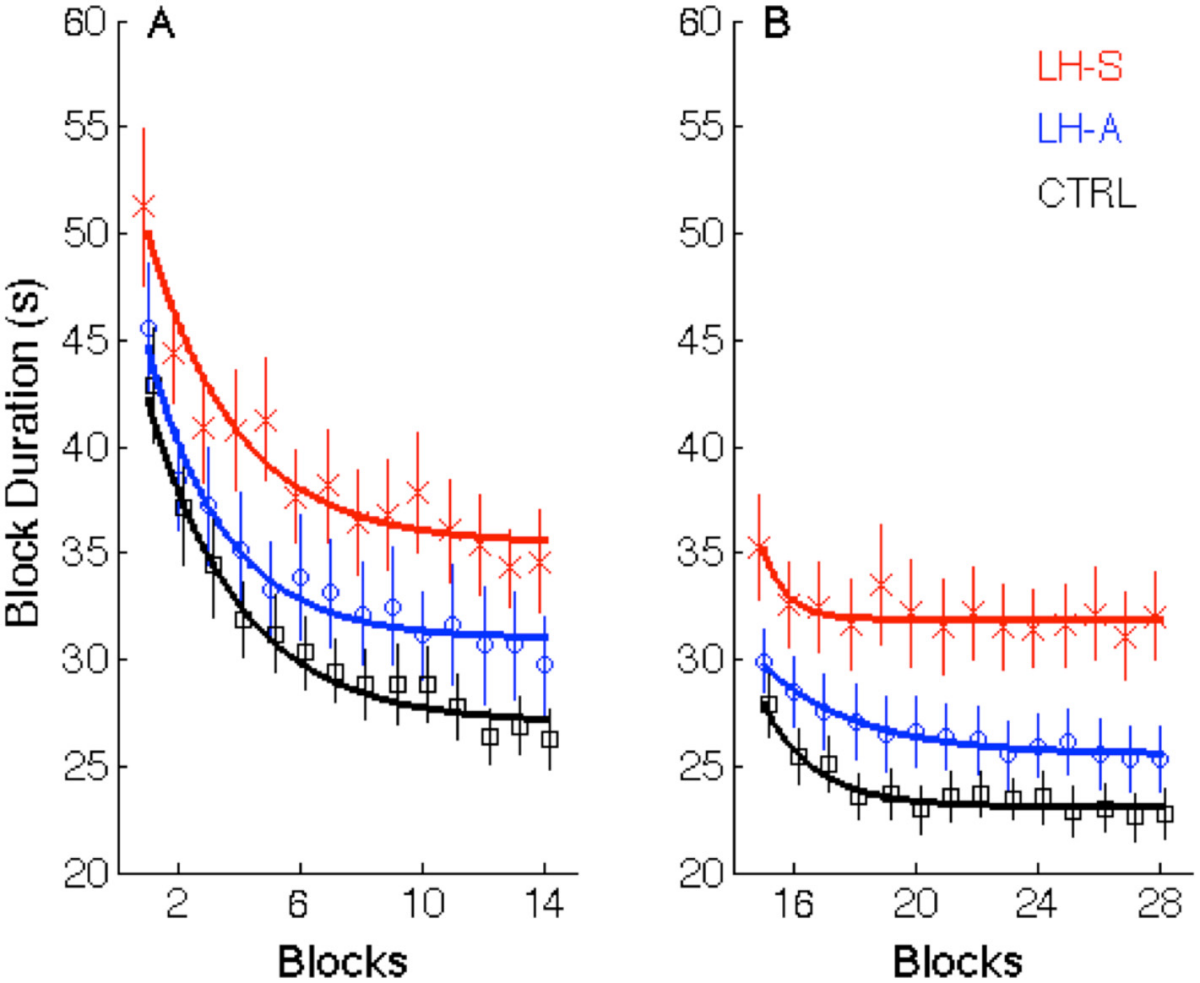
Block Duration in Session 1 (A) and Session 2 (B). Data points represent group means for each block and error bars depict standard errors. Black squares = healthy controls; blue circles = left hand asymptomatic (LH-A) patients with PD; red crosses = left hand symptomatic patients (LH-S) with PD. Thick solid lines represent group-averaged trajectories based on a single exponential fit.

Block Duration for the initial learning session is depicted in Fig 2A. A 3 (Group) x 14 (Block) ANCOVA with age as the covariate revealed a significant Block main effect (F_(13,884)_ = 63.31; p<0.001) as the time to complete a block of practice decreased as a function of training. There was also a significant Group main effect (F_(2,67)_ = 3.90; p = 0.025). Follow-up analyses indicated that the LH-S patients with PD were significantly slower relative to the healthy controls (F_(1,44)_ = 11.08; p = 0.002). The Block x Group interaction was not significant (F_(26,884)_ = 0.51; p = 0.98), indicating that the rate of change in movement speed did not differ among the three groups in the initial learning session (although see analyses of Performance Index below).

#### 3.1.2. Movement Accuracy

**Fig 3:**
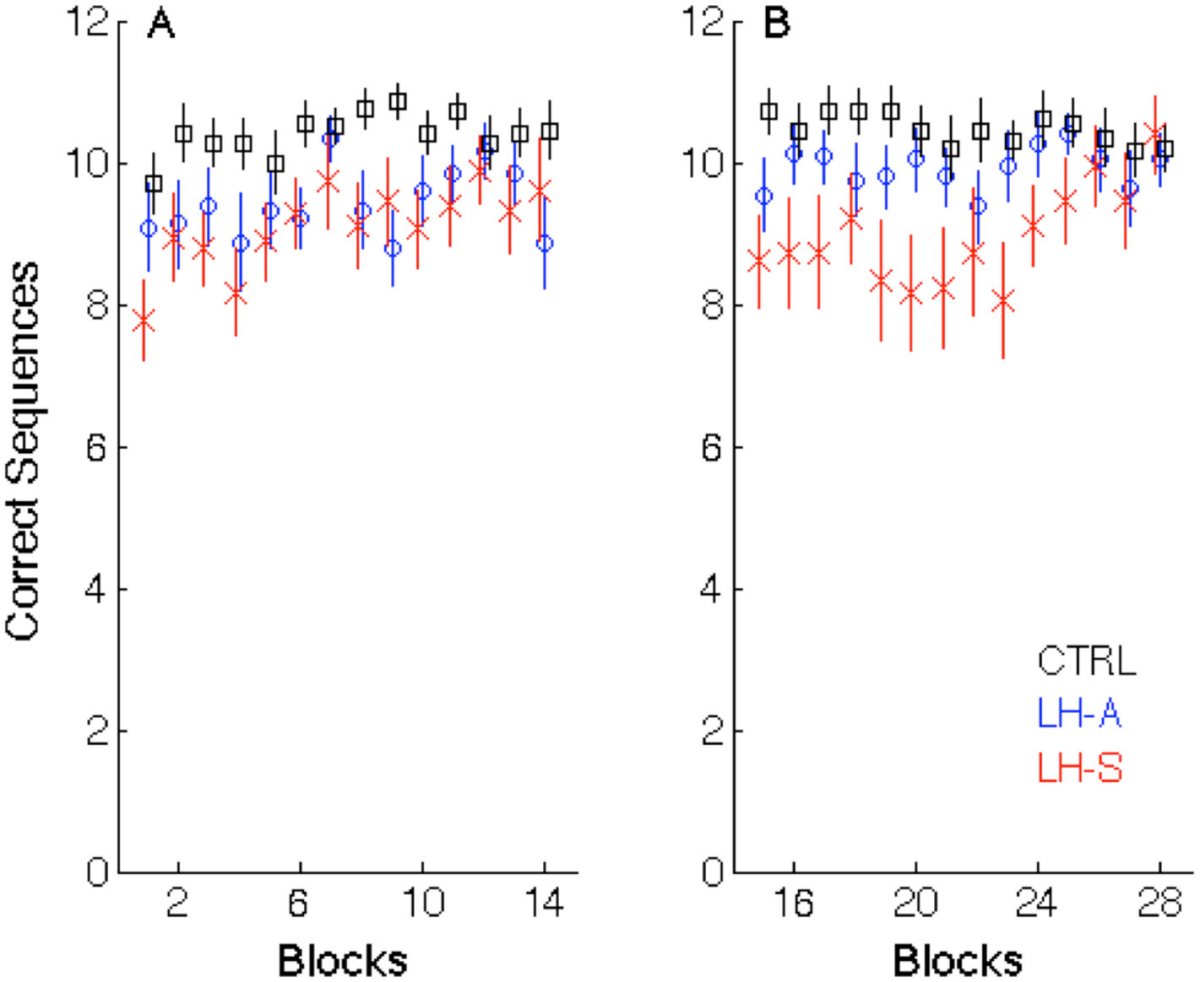
The number of correct sequences in Session 1 (A) and Session 2 (B). Data points represent group means for each block and error bars depict standard errors. Black squares = healthy controls; blue circles = left hand asymptomatic (LH-A) patients with PD; red crosses = left hand symptomatic patients (LH-S) with PD.

The number of correct sequences completed in the initial learning session is depicted in Fig 3A. The 3 (Group) x 14 (Block) ANCOVA indicated a significant Block main effect (F_(13,884)_ = 8.08; p<0.001), as movement accuracy was modulated by practice. Results also revealed a significant Group main effect (F_(2,67)_ = 4.28; p = 0.018) and subsequent analyses indicated that the healthy controls were more accurate relative to the LH-A (F_(1,50)_ = 6.34; p = 0.015) and LH-S patients (F_(1,44)_ = 8.33; p = 0.006). Similar to the analysis of movement speed above, the Block x Group interaction was not significant (F_(26,884)_ = 1.18; p = 0.25).

#### 3.1.3. Performance Index

**Fig 4:**
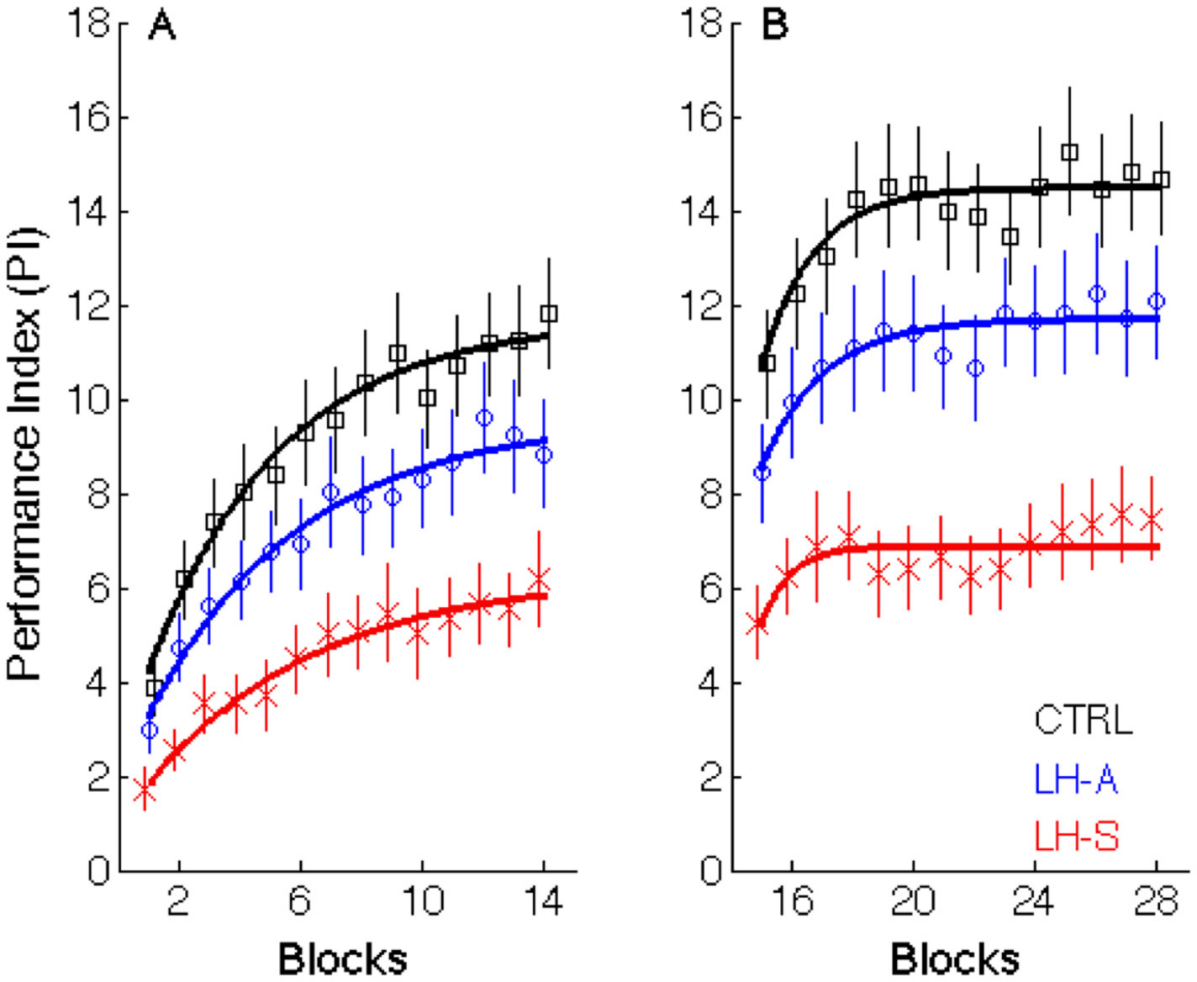
Performance Index (PI) in Session 1 (A) and Session 2 (B). Data points represent group means for each block and error bars depict standard errors. Black squares = healthy controls; blue circles = left hand asymptomatic (LH-A) patients with PD; red crosses = left hand symptomatic patients (LH-S) with PD. Thick solid lines represent group-averaged trajectories based on a single exponential fit.

As both movement speed and accuracy were modulated by practice, we computed an aggregate speed / accuracy dependent measure (PI; depicted in Fig 4A). Results from the 3 (Group) x 14 (Block) ANCOVA with age as the covariate revealed a significant Block main effect (F_(13,884)_ = 51.4; p<0.001) as PI increased as a function of practice. A significant Group main effect was also evident (F_(2,67)_ = 7.57; p = 0.001) and follow-up analyses indicated that performance of the LH-S patients with PD was significantly worse relative to both the LH-A patients (F_(1,39)_ = 4.92; p = 0.032) and the healthy controls (F_(1,44)_ = 16.00; p<0.001). Interestingly, there was a trend for a difference between the LH-A and controls (F_(1,50)_ = 3.63; p = 0.063), a result that can be attributed predominantly to a deficit in movement accuracy. The Block x Group interaction was marginally significant (F_(26,884)_ = 1.45; p = 0.07), indicating a trend for a difference among the three groups in the rate of performance improvement. Follow-up analyses were conducted to decompose this effect; however, as the initial ANCOVA only indicated a trend for a significant effect, significance thresholds for the follow-up contrasts were Bonferroni corrected to a value of 0.017 (e.g., 0.05 / 3). Results revealed that the PI improvement in the LH-S patients was significantly different compared to the healthy controls (F_(13,585)_ = 2.25; p = 0.007), suggesting impaired learning in the LH-S patients. By contrast, the LH-A patients did not differ from the LH-S patients or the healthy controls (p > 0.18) with respect to the rate of PI improvement in Session 1.

One could make the case that the initial learning session consists of more than one learning phase. Specifically, a visual inspection of Fig 4 reveals substantial, rapid improvements over the first 4 blocks approximately followed by a more gradual improvement over the last 10 blocks. To better understand this initial learning session, we conducted additional analyses on these subsets of blocks. A 3 (Group) x 4 (Block) ANCOVA on the first 4 blocks revealed a significant interaction (F_(6,204)_ = 2.66; p = 0.017). Follow-up analyses indicated that there was no difference between the LH-A patients and healthy controls. Conversely, the LH-S patients had a decreased rate of improvement relative to the healthy controls (F_(3,135)_ = 4.94; p = 0.003), indicating impaired learning over the first 4 blocks. The LH-S patients were also significantly different, collapsed across the 4 blocks of practice, from both the LH-A patients (F_(1,39)_ = 4.82; p = 0.034) and the controls (F_(1,44)_ = 12.86; p<0.001). An analysis of the last 10 blocks of training indicated no differences among the three groups in the rate of performance improvement (F_(8,272)_ = 1.03; p = 0.41). However, the LH-S patients were significantly worse, collapsed across blocks of practice, than the controls (F_(1,44)_ = 13.74; p<0.001) and there was a trend for a difference relative to LH-A patients (F_(1,39)_ = 3.95; p = 0.054). Consistent with the analyses of all 14 blocks of Session 1, there was also a trend for a group difference between the LH-A patients and healthy controls in the last 10 blocks (F_(1,50)_ = 3.77; p = 0.058).

Collectively, the results from the initial training session revealed that when both speed and accuracy components were taken into account, the LH-S patients with PD exhibited significant learning and performance deficits. These impairments were evident as early as the first 4 blocks of practice. Conversely, the LH-A patients were statistically comparable to the healthy controls across the first 4 blocks of practice. There was, however, a trend for a group difference between these two groups collapsed across the last 10 blocks of practice, suggesting that impairments in the LH-A patients with PD emerge as a function of training on the MSL task.

### 3.2. Retest Session

#### 3.2.1. Movement Speed

Block Duration for the retest is depicted in Fig 2B. A 3 (Group) x 14 (Block) ANCOVA revealed a significant Block main effect (F_(13,884)_ = 16.38; p<0.001) as the time to complete a block of practice continued to decrease as a function of the extended practice. There was also a significant Group main effect (F_(2,67)_ = 8.29; p<0.001). Follow-up analyses indicated that the LH-S patients with PD were significantly slower as compared to both the healthy controls (F_(1,44)_ = 19.15; p<0.001) and the LH-A patients (F_(1,39)_ = 4.39; p = 0.043). Interestingly, there was a trend for a difference between LH-A patients and healthy controls (F_(1,50)_ = 3.06; p = 0.086). The Block x Group interaction was not significant (F_(26,884)_ = 0.88; p = 0.64), demonstrating that the rate of change in movement speed did not differ among the three groups in the extended training phase.

#### 3.2.2. Movement Accuracy

The 3 (Group) x 14 (Block) ANCOVA indicated a significant Group by Block interaction (F_(26,884)_ = 1.55; p = 0.040), indicating the modulation of accuracy as a function of blocks in Session 2 differed among the three groups. Follow-up analyses were conducted to decompose this effect and results revealed that the LH-S patients and healthy controls differed in this modulation (F_(13,585)_ = 2.21; p = 0.008), an effect that appears to be largely driven by the sharp increase in accuracy exhibited by the LH-S patients at the end of Session 2. Additionally, there was a trend for a group difference in accuracy in the retest between the LH-A patients and healthy controls (F_(1,50)_ = 3.05; p = 0.087).

#### 3.2.3. Performance Index

Similar to the initial learning session, we conducted analyses on the combined speed / accuracy measure PI during the retest (Fig 4B). Significant Block (F_(13,884)_ = 11.5; p<0.001) and Group (F_(2,67)_ = 12.27; p<0.001) main effects were revealed. Follow-up analyses for the Group main effect indicated that performance of the LH-S patients was again significantly worse compared to both the LH-A patients (F_(1,39)_ = 8.27; p = 0.007) and the healthy controls (F_(1,44)_ = 27.11; p<0.001). Importantly, however, performance of the LH-A patients was also significantly worse than that of the healthy older adults (F_(1,50)_ = 5.12; p = 0.028). The Group by Block interaction in Session 2 was not significant (p = 0.63).

### 3.3. Offline Changes

Differences between the two MSL sessions (mean of the last 2 blocks of Session 1 subtracted from the mean of the first 2 blocks of Session 2) were taken as an indicator of motor sequence memory consolidation. A one-way ANCOVA with age as the covariate was conducted for each dependent measure (speed, accuracy and PI) and the offline changes were comparable for the three groups of participants (all p>0.24), indicating that offline consolidation processes did not differ. To demonstrate that the choice of using 2 blocks in the computation of offline changes did not influence the results, we conducted a similar analysis but based on the last 4 blocks of Session 1 and the first 4 blocks of Session 2 [9,47]. Again, the changes for the three groups remained comparable (all p>0.12), confirming the lack of a deficit in motor sequence consolidation in patients with PD.

## Discussion

Results from the current study demonstrate that LH-S *de novo* patients with PD demonstrated significant impairments during both initial learning and extended practice, whereas deficits emerged in the later learning stages in the LH-A patients. Critically, the impairment in extended practice in the LH-A patients was evident even before the appearance of traditional clinical symptoms in the tested hand.

Although previous behavioral research investigating MSL in PD has been largely inconsistent with respect to whether patients with PD demonstrate learning deficits [10–22], our results from the LH-S patients suggest that the disease-related alterations in relevant corticostriatal networks shortly following the appearance of clinical symptoms in the tested hand are severe enough to affect both the initial learning and extended practice phases. As associative and sensorimotor regions of the striatum are more involved in the early and later stages of learning, respectively [29,48,49], it is thus likely that the disease-related dopaminergic denervation extends into both striatal regions [29]. Critically, this explanation would explain not only the deficits in both sessions of the MSL task, but also the impairments in motor function evaluated by the UPDRS (Table 1). Yet as the current study did not directly examine striatal degradation in the patients with PD using PET for example, this explanation certainly awaits further investigation.

The LH-A patients performed worse compared to the healthy controls in the retest session of the MSL task, suggesting that the disease-related dopaminergic denervation was robust enough to trigger deficits during extended practice of the sequence task. This deficit was even evident prior to the appearance of traditional clinical symptoms in the tested hand. It should be emphasized that—unlike the LH-S patients in Session 1—the rate of performance improvement in Session 2 did not differ between LH-A patients and controls. Rather, the LH-A patients exhibited worse performance collapsed across the blocks of practice. We contend that this reflects a deficit in the later learning phases. Since the magnitude of improvements is smaller in the later learning stages, it is possible that deficits are reflected by decreases in the asymptotic levels obtained as opposed to decreases in learning rate. Similar to the LH-S patients, the impairment in Session 2 exhibited by the LH-A patients with PD may again be explained by the disease-related progression in the striatum, and the putamen in particular. As the sensorimotor regions of the striatum are more critical for later stages of learning [29,48,49], it follows that the LH-A patients with PD experience deficits in the later, extended training stage.

**Fig 5:**
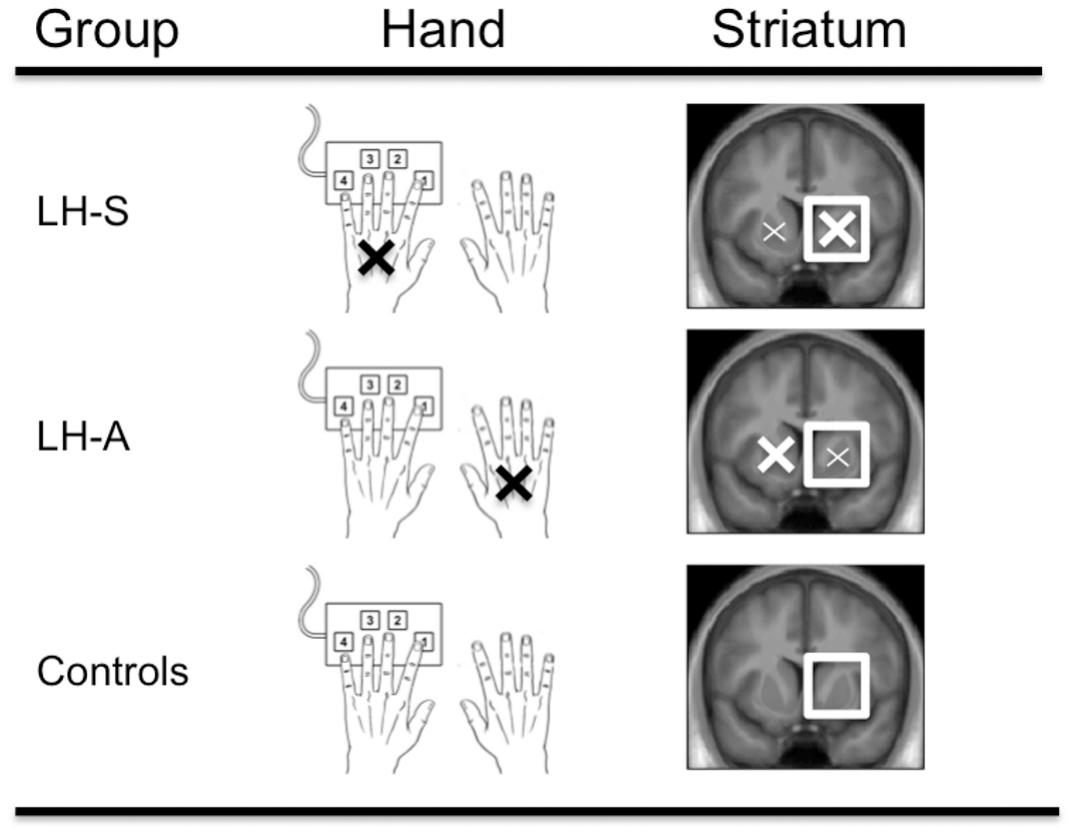
Schematic of how performance on the MSL task may reflect disease progression. All participants completed the MSL task with their non-dominant left hand. X’s denote the affected side and the size of the X represents the relative magnitude of the impairment. Squares indicate that the right (contralateral) striatum is more critical for the execution of the MSL task since the left hand was used. In the left hand symptomatic (LH-S) group, the MSL task targeted the more affected right striatum. In contrast, in the left hand asymptomatic (LH-A) group, motor deficits were limited to the right side; thus, the MSL task targeted the less affected right striatum. In the control group, the MSL task targeted the intact right striatum. Comparison of the experimental groups may represent progression of PD. Please note that this schematic is intended to depict the laterality aspect of the research approach and does not address changes within striatal subregions as a function of MSL training. These details are discussed in the main text.

Bridging the results from the two groups, we propose that the pattern of results may be explained by the severity of the disease within the hemisphere-specific striatal regions that are critical for the MSL task (Fig 5). Specifically, as the onset of motor symptoms in patients with PD is predominantly asymmetric, the dopaminergic deficiency in one hemisphere is more robust than the other [50]. Thus, asking participants to complete a MSL paradigm with the symptomatic or asymptomatic hand relies on the more or less affected contralateral corticostriatal network, respectively. Thus, in the context of the current study, the LH-S patients can be conceptualized as a model for more advanced PD—relative to the LH-A patients—as the dopaminergic deficiency in the right (contralateral) striatum was presumably *more* marked. Conversely, the LH-A patients can be conceptualized as in earlier disease stages as the dopaminergic deficiency in the right (contralateral) striatum was presumably *less* severe. Accordingly, the deficits in both initial learning and extended practice in the LH-S patients may be the result of advanced disease progression in the right (contralateral) striatum. For example, and as highlighted above, striatal denervation in this group likely extends beyond the sensorimotor territories that are affected early in the disease process and into the associative regions. Conversely, the impairments that emerge later in the learning process in the LH-A patients are the result of a less advanced stage of disease, as the striatal denervation is more limited to the sensorimotor regions of the striatum. The associative regions of the contralateral striatum would then be less affected in these patients, as the LH-A patients were more similar to healthy controls early in the learning process, and the first 4 blocks of practice in particular during which rapid behavioral improvements are evident.

Although this explanation is conjectural and awaits additional investigations, it does offer several interesting predictions to be tested in future research. As extended practice on the MSL task involves regions of the striatum that show some of the earliest disease-related dopaminergic degradations [29–31], then deficits in “true” prodromal patients (i.e., prior to the appearance of any clinical symptoms in either hand) may not emerge until even later in the learning process than examined in the current study. Similarly, if the paradigm were extended to include additional MSL training sessions, it could be predicted that the behavioral deficits observed in the patients would increase in magnitude. Indirect support from this latter prediction comes from an examination of performance in Figs 2 and 4. The performance differences between the three groups appear to increase as a function of blocks of practice.

The deficits in Session 2 demonstrated by both patient groups is consistent with previous research in which impairments in performing motor sequences emerged only after continued training [21,25,27]. Such results have been attributed to difficulties in ‘automatizing’ the previously learned sequences [26,51], a function that has indeed been attributed to the basal ganglia [52]. Although results from the current study cannot speak to ‘automatization’ as 28 blocks of training across two testing days is likely not sufficient to reach this learning phase, our findings do indicate that *de novo* patients with PD have deficits in the extended training phase.

Admittedly, it is likely that the performance of the LH-S patients can partially be attributed to execution—as opposed to learning—deficits, as these patients demonstrated impairments with the left hand during the clinical testing. However, our results suggest that LH-S patients also demonstrated a deficit in learning, as indicated by the significant Group x Block interaction as compared to the control group in Session 1 (F_(13,585)_ = 2.25; p = 0.007). Furthermore, we contend that it is unlikely that the impairments observed in the LH-A patients can be explained *purely* by motor execution deficits. First, these patients did not demonstrate any impairment with the left hand on the clinical screening. Second, if we use the first block of the first session on the MSL task as a measure of motor execution (i.e., prior to any substantial learning), the LH-A patients and the controls did not differ on movement speed, accuracy or the aggregate measure of PI (all p>0.17). This then suggests that the deficits evident in the LH-A patients emerged as a function of practice on the MSL task and are not simply the result of pure execution difficulties.

Memory consolidation, as assessed by the offline changes in performance, did not differ among the three groups. This finding is consistent with a recent study that demonstrated patients with PD and healthy older adults showed comparable overnight stabilization of a motor sequence memory trace [21]. Thus, memory consolidation appears unaffected in *de novo* patients with PD. This result may not be that surprising as previous studies have shown that even healthy older adults have significant impairments in offline consolidation despite comparable initial learning [53–56]. As inter-session differences in healthy controls typically approximate zero, differences between groups will only be detected if patients with PD demonstrate substantial *deterioration* of the memory trace (e.g., forgetting), which was not the case in this study or in earlier research [21].

## Conclusion

Our results collectively suggest that *de novo* patients with PD demonstrate deficits in the initial and extended—depending on disease severity—motor sequence learning phases, whereas consolidation processes remain unaffected. Future research should employ neuroimaging approaches in order to provide a direct link between behavioral deficits and disease-related degradation in relevant cortico-striatal networks. Moreover, these results should be extended by examining individuals with a genetic predisposition to PD and expanding the sequence learning paradigm in order to facilitate the classification (i.e., healthy control, symptomatic PD, asymptomatic PD) based on individual MSL performance. Results would then provide insights into whether performance on a MSL task may serve as a non-invasive and inexpensive marker of Parkinson’s disease and its progression.

## Supporting Information

S1 Table Group means and SD for each block of the MSL task. (PDF)
