# Supplementary material for: Motor Sequence Learning and Consolidation in Unilateral De Novo Patients with Parkinson’s Disease
Source: PLoS One. 2015 Jul 29;10(7):e0134291. doi: 10.1371/journal.pone.0134291 (PMC4519305; doi:10.1371/journal.pone.0134291)
Supplement: S1 Table — (PDF) [file pone.0134291.s001.pdf]

Supporting Information

Supporting Table 1. Group means and SD for each block of the MSL task.

	LH-S	LH-A	Controls
**Session 1**			
Block 1	1.73 (1.93)	2.99 (2.49)	3.85 (3.17)
Block 2	2.55 (1.81)	4.73 (3.60)	6.16 (4.31)
Block 3	3.53 (2.58)	5.61 (3.90)	7.38 (4.95)
Block 4	3.54 (2.60)	6.15 (4.08)	8.03 (5.31)
Block 5	3.71 (3.09)	6.77 (4.11)	8.38 (5.48)
Block 6	4.48 (3.08)	6.92 (4.54)	9.30 (6.04)
Block 7	5.00 (3.78)	8.03 (5.66)	9.54 (5.92)
Block 8	5.06 (3.22)	7.75 (5.00)	10.35 (5.89)
Block 9	5.46 (4.35)	7.91 (5.05)	10.97 (6.92)
Block 10	5.02 (3.99)	8.31 (5.13)	10.00 (5.50)
Block 11	5.36 (3.49)	8.66 (5.31	10.72 (5.65)
Block 12	5.64 (3.54)	9.61 (5.75)	11.17 (5.79)
Block 13	5.55 (3.23)	9.21 (5.81)	11.23 (6.20)
Block 14	6.20 (4.22)	8.83 (5.60)	11.79 (6.24)
**Session 2**			
Block 1	5.25 (3.22)	8.43 (4.98)	10.75 (6.10)
Block 2	6.22 (3.38)	9.94 (5.65)	12.23 (6.17)
Block 3	6.86 (4.83)	10.66 (5.58)	13.04 (6.53)
Block 4	7.09 (3.91)	11.07 (6.44)	14.23 (6.48)
Block 5	6.29 (3.78)	11.43 (6.22)	14.51 (6.91)
Block 6	6.41 (3.68)	11.40 (6.04)	14.57 (6.37)
Block 7	6.64 (3.73)	10.91 (5.35)	13.99 (6.73)
Block 8	6.25 (3.45)	10.66 (5.34)	13.84 (6.22)
Block 9	6.42 (3.59)	11.83 (5.50)	13.47 (5.52)
Block 10	6.90 (3.62)	11.65 (5.63)	14.51 (6.74)
Block 11	7.18 (4.15)	11.84 (6.27)	15.25 (7.28)
Block 12	7.34 (4.00)	12.22 (6.22)	14.43 (6.33)
Block 13	7.56 (4.23)	11.72 (5.97)	14.81 (6.57)
Block 14	7.47 (3.75)	12.05 (5.73)	14.68 (6.33)
